# High-Risk Polymorphisms Associated with the Molecular Function of Human HMGCR Gene Infer the Inhibition of Cholesterol Biosynthesis

**DOI:** 10.1155/2022/4558867

**Published:** 2022-06-06

**Authors:** Keshob Chandra Das, Mohammad Uzzal Hossain, Md Moniruzzaman, Md Salimullah, Sharif Akhteruzzaman

**Affiliations:** ^1^Molecular Biotechnology Division, National Institute of Biotechnology, Ganakbari, Ashulia, Savar, Dhaka-1349, Bangladesh; ^2^Bioinformatics Division, National Institute of Biotechnology, Ganakbari, Ashulia, Savar, Dhaka-1349, Bangladesh; ^3^Department of Pharmacology, Medical Sciences Division, University of Oxford, Oxford OX12JD, UK; ^4^Department of Genetic Engineering and Biotechnology, University of Dhaka, Dhaka, Bangladesh

## Abstract

HMG-CoA reductase or HMGCR (3-hydroxy-3-methylglutaryl-CoA reductase) is a rate-limiting enzyme involved in cholesterol biosynthesis. HMGCR plays an important role in the possible occurrence of hypercholesterolemia leading to atherosclerosis and coronary heart disease. This enzyme is a major target for cholesterol-lowering drugs such as “statin” which blocks the synthesis of mevalonate, a precursor for cholesterol biosynthesis. This study is aimed at characterizing deleterious mutations and classifying functional single nucleotide polymorphisms (SNPs) of the HMGCR gene through analysis of functional and structural evaluation, domain association, solvent accessibility, and energy minimization studies. The functional and characterization tools such as SIFT, PolyPhen, SNPs and GO, Panther, I-Mutant, and Pfam along with programming were employed to explore all the available SNPs in the HMGCR gene in the database. Among 6815 SNP entries from different databases, approximately 388 SNPs were found to be missense. Analysis showed that seven missense SNPs are more likely to have deleterious effects. A tertiary model of the mutant protein was constructed to determine the functional and structural effects of the HMGCR mutation. In addition, the location of the mutations suggests that they may have deleterious effects because most of the mutations are residing in the functional domain of the protein. The findings from the analysis predicted that rs147043821 and rs193026499 missense SNPs could cause significant structural and functional instability in the mutated proteins of the HMGCR gene. The findings of the current study will likely be useful in future efforts to uncover the mechanism and cause of hypercholesterolemia. In addition, the identified SNPs of HMGCR gene could set up a strong foundation for further therapeutic discovery.

## 1. Introduction

Hypercholesterolemia, or elevated blood cholesterol levels, is a condition associated with an increased risk of atherosclerosis and coronary heart disease [1]. These cardiovascular diseases are the result of elevated serum non-high-density lipoprotein cholesterol (non-HDLC) levels. The variations in non-HDL cholesterol among individuals are a result of disparities among the genes involved in the biosynthesis of cholesterol as well as environmental factors such as diet and lifestyle [2–4]. Cholesterol is one of the three major classes of lipids; the complex procedure of cholesterol biosynthesis is essential for all animal life. The process of cholesterol biosynthesis begins with the mevalonate pathway, and the rate-limiting enzyme of this pathway is called 3-hydroxy-3-methyl-glutaryl-coenzyme A reductase (HMG-CoA reductase or HMGCR) [5, 6].

HMGCR is regulated via a negative feedback mechanism mediated by sterols and nonsterol metabolites derived from mevalonate. Normally, cholesterol produced from the internalization and degradation of low-density lipoprotein (LDL) via the LDL receptor suppresses the activity of this enzyme in mammalian cells. The expression of LDL receptors in the liver is induced by competitive inhibitors of the reductase, which in turn upsurges the catabolism of plasma LDL and lessens the plasma concentration of cholesterol, an important factor of atherosclerosis [7–11]. Thus, HMGCR serves as a target for the cholesterol-lowering drug, statin. Statins are a class of drugs that act as competitive inhibitors of the enzyme. The inhibition of the enzyme by the drug reduces the rate by which HMGCR can produce mevalonate, the next molecule in the cascade that eventually produces cholesterol. This is significant because most circulating cholesterol comes from internal manufacture rather than a diet. When the liver can no longer produce cholesterol, the liver cells begin to express LDL receptors on their surface, which bind LDL cholesterol particles and internalize them and the levels of cholesterol in the blood will fall [12]. Despite the fact that statin medicines are effective at decreasing cholesterol, the patient's response to them varies greatly among individuals [13, 14]. Several pharmacogenetics investigations and genome-wide association studies (GWAS) have revealed genetic variants in genes involved in cholesterol homeostasis as a significant contributor of the diversity in statin response reported among patients [15–17]. These studies have shown that the SNPs (single nucleotide polymorphisms) are related to not only the therapeutic effect of statin but also blood lipid level, consequently the risk of developing cardiovascular disease [18–20].

Functional genomics and mutational analysis with the advancement of bioinformatics tools have already provided the key advances in disease diagnosis, prognosis, and therapeutic efficacy [21–35]. The mutational effect upon the protein structure and disease prognosis has been identified by the in silico techniques which further could be validated by the wet lab research [36–42]. However, the in silico analysis of coding SNPs of the HMGCR gene is still yet to be explored to observe the mutational effect. Among which missense coding SNPs are thought to have the principal impact on phenotype and may exert deleterious effects on the structure, function, solubility, or stability of proteins. Hence, this investigation is aimed at characterizing the deleterious mutations of HMGCR gene. Our study involved the following: (a) retrieval of SNPs in the HMGCR gene from available databases, (b) detecting deleterious missense SNPs that can change splicing and gene expression patterns using sequence- and structure-based homology search, (c) predicting the precise effects of the substitutions of amino acids on secondary structures via solvent accessibility and stability of the structure, and (d) prediction of change in the domain constructions due to the mutations. This study is the first extensive in silico analysis of the HMCGR gene and expected to establish a strong foundation for structure-function relationship of the enzyme and population-specific variation studies in years to come. Further, it might explore the specific mutation-based drug discovery against hypercholesterolemia.

## 2. Materials and Methods

### 2.1. Retrieval of SNP Datasets

SNP of the HMGCR gene and their protein sequences (FASTA format) were retrieved from the dbSNP database (http://www.ncbi.nlm.nih.gov/SNP/) for computational analysis. Various filters were used for the selection of SNPs related to HMGCR, such as missense, inframe deletion, inframe insertion, initiator codon variant, and synonymous.

### 2.2. Analysis of Functional Consequences of Missense SNPs

Sorting Intolerant from Tolerant (SIFT) [43] is an algorithm that predicts the potential impact of amino acid substitutions on protein function. This program assumes that evolutionarily conserved regions tend to be less tolerant of mutations, and hence, amino acid substitutions or insertions/deletions in these regions are more likely to affect function [43]. SIFT considers the composition of amino acids and calculates the score after searching a query protein against a protein database to obtain homologous protein sequences. All the retrieved mutations were submitted to this tool. A SIFT score ranges from 0 to 1; it is a standardized probability of perceiving the new amino acid at that position. It also calculates the tolerance index (TI) of a particular amino acid substitution. SIFT score is classified as tolerant (ranging from 0.201 to 1.00) or intolerant (0.051–0.10) and borderline (0.101–0.20). Hence, an SNP's functional consequence is inversely proportional to the tolerance index (TI) [44]. In this study, the reference SNP cluster identification (rsID) of each SNP of human HMGCR gene obtained from NCBI was submitted as a query sequence to SIFT for homology searching. The SIFT score of ≤0.05 indicates the deleterious effect of missense variants on protein function.

### 2.3. Functional Consequences of Missense SNPs by Structural Homology

To comprehend the functional significance of a protein, it is essential to scrutinize the damaged coding missense SNPs at the structural level. Polymorphism Phenotyping-2 or PolyPhen-2 [45] is an automatic tool for the prediction of the possible impact of an amino acid substitution on the structure and function of a human protein. To understand the functional consequence, the protein sequence of our gene along with mutational position and two amino acid variants was submitted as the query. The PolyPhen-2 calculates the posterior probability that a missense SNP is damaging by a Bayesian classifier [46]. The conservation of a position in the MSA and the deleterious effect on the protein structure results in the Position-Specific Independent Count (PSIC) score that ranges from 0 to 1 [45]. The categorization of the missense SNPs results in possibly damaging and probably damaging (PSIC > 0.5) or benign (PSIC < 0.5). PolyPhen-2 predicts the possible functional impact of the SNPs on structure based on the difference of the PSIC score.

### 2.4. Characterization of Functional Missense SNPs

SNP&GO (Gene Ontology) [47, 48] was used for the characterization of functional missense SNPs. The impact of protein variations was predicted via the SNP&GO algorithms, using functional information categorized by GO terms of the three main roots: molecular function, biological process, and cellular component. The selected SNPs were submitted into this tool to determine the functional insights. The sequence profile is then calculated by performing one run of BLAST against the UniRef90 dataset to select homologous sequences with an *E* value lower than 10^−9^. The algorithm uses a support vector machine (SVM) based analyzing method, which includes the sequence environment of the variation and a log-odd score calculated considering all the Gene Ontology terms connected to the mutated protein and their parents in the GO graph. A probability score higher than 0.5 reveals the disease-related effect of mutation on the protein function [48].

PANTHER cSNP (Protein ANalysis Through Evolutionary Relationship coding_SNP), the most popular online protein fold identification server, calculates the likelihood of a single AA change on protein function, and it is based on the PANTHER-PSEP (Position_Specific Evolutionary Preservation) method [49]. PMUT is based on the use of different kinds of sequence information to label mutations and neural networks to process this information. FASTA sequence was inputted, and result was based on the differences among disease related and neutral variations of protein sequence. Probability score higher than 0.5 reveals the disease-related effect of mutation on the protein function [50]. MutPred2 is a machine learning-based method and software package that integrates genetic and molecular data to reason probabilistically about the pathogenicity of amino acid substitutions [51]. MutPred2 was utilized to predict the pathogenicity of all the nsSNPs of the HMGCR gene. Finally, SNAP-2 uses neural networks to anticipate the impact of single amino acid substitutions (SNPs). Prediction scores are visualised as a heat map [51].

### 2.5. Prediction of Modification in Stability upon Mutation

To predict the change in stability owed to mutations, the I-Mutant 2.0 server was used. This is a support vector machine (SVM) based tool server, which analyzes the structure or the sequence of the protein. I-Mutant 2.0 is a classifier that can automatically predict the change in protein structural stability upon mutations, and it also acts as a regression estimator which predicts the change in Gibbs free energy differences between the mutated and wild-type protein in kcal/mol [52].

### 2.6. Missense SNP in Functional Region

The Pfam server was used to discern the location of missense SNP into the functional region in protein structure. This is a database of protein families, their annotations, and multiple sequence alignments, which provides a complete and accurate classification of protein families and domains [53]. The identification of domains that occur within proteins can provide insights into their function; thus, Pfam was to analyze the functional region of the HMGCR.

### 2.7. Modeling of the Mutated Protein

Then, a Virtual Mutation (VM) procedure was applied to substitute amino acids in the atomic models [54]. Accelrys Discovery Studio 4.0 was then used to generate a mutated sequence for the corresponding amino acid substitutions [55]. The regenerated mutant sequences were used further for mutant modeling, which was performed through HHpred Modeller [56]. Later, the InterEvDock2 server was used to calculate the degree of evolutionary conservation at each amino acid position of the HMGCR protein [56]. HHpred chooses the best suitable template and generates a protein model through successive steps, such as profile construction, similarity analysis, and structural properties. Then, the Swiss model also confirmed the same template as HHpred Modeller selected [57]. A rigorous manner of protein modeling was nominated to get a perfect model.

### 2.8. Location and Structural Conformation of Mutations

We have utilized the STRIDE server to predict the mutational site upon the 3D structure. This server offers an interactive interface to the secondary structure assignment program STRIDE [58].

### 2.9. Molecular Dynamic Simulation

In order to evaluate the impact of the SNPs rs147043821 and rs193026499 on the stability of HMGCR gene product under physiological conditions, 50 ns molecular dynamic simulation was performed in using GROMACS (version 2021.1). The GROMOS9643a1 force field was applied on the protein-ligand complex [59]. The physiological condition of the system was defined (300 K, pH 7.4, 0.9% NaCl). The structures were solvated in a dodecahedral box of the SPC (simple point charge) water model with its edges at 1 nm distance from the protein surface. The overall charge of the system was neutralized using the genion module. Energy minimization of the neutralized system was carried out using the steepest descent minimization algorithm with maximum number of minimization steps to perform set at 50000. The ligand was restrained before carrying out the isothermal-isochoric (NVT) equilibration of the system for 100 ps with short-range electrostatic cutoff value of 1.2 nm. Isobaric (NPT) equilibration of the system was carried out for 100 ps following the NVT with short-range van der waals cutoff fixed at 1.2 nm. Finally, a 50 ns molecular dynamic simulation was run using periodic boundary conditions and time integration step of 2 fs. The energy of the system was saved every 100 ps. For calculating the long-range electrostatic potential, the Particle Mesh Ewald (PME) method was applied. Short-range van der waals cutoff was kept at 1.2 nm. The modified berendsen thermostat was used to control simulation temperature while the pressure was kept constant using the Parrinello-Rahman algorithm. The simulation time step was selected as 2.0 fs. The snapshot interval was set to 100 ps for analyzing the trajectory data. Finally, all of the trajectories were concatenated to calculate and plot root mean square deviation (RMSD), root mean square fluctuation (RMSF), radius of gyration (Rg), and solvent accessible surface area (SASA) data.

### 2.10. Data Analytics and Presentation

Figures and tables were used to present the results of our investigation. Based on the information from our results, the figures and tables were created using Excel, PowerPoint, and BioRender. The tools were also used to create the majority of the figures.

## 3. Results

**Figure 1:**
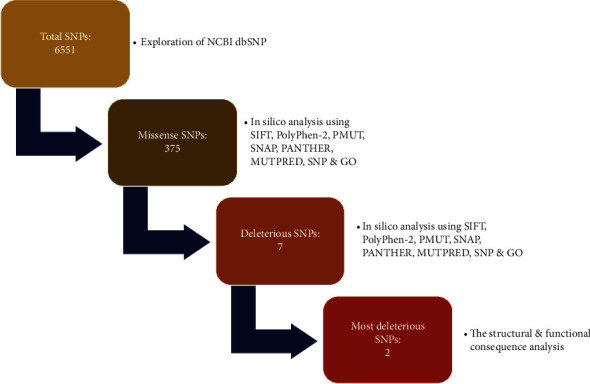
Flow diagram of the overall work.

The flowchart illustrates the overall procedure of identification and categorization of detrimental SNPs in HMGCR along with the structural and functional consequence analysis upon mutation (Figure 1).

### 3.1. HMGCR Gene Is Prone to Point Mutation and Rich in Missense Type

**Figure 2:**
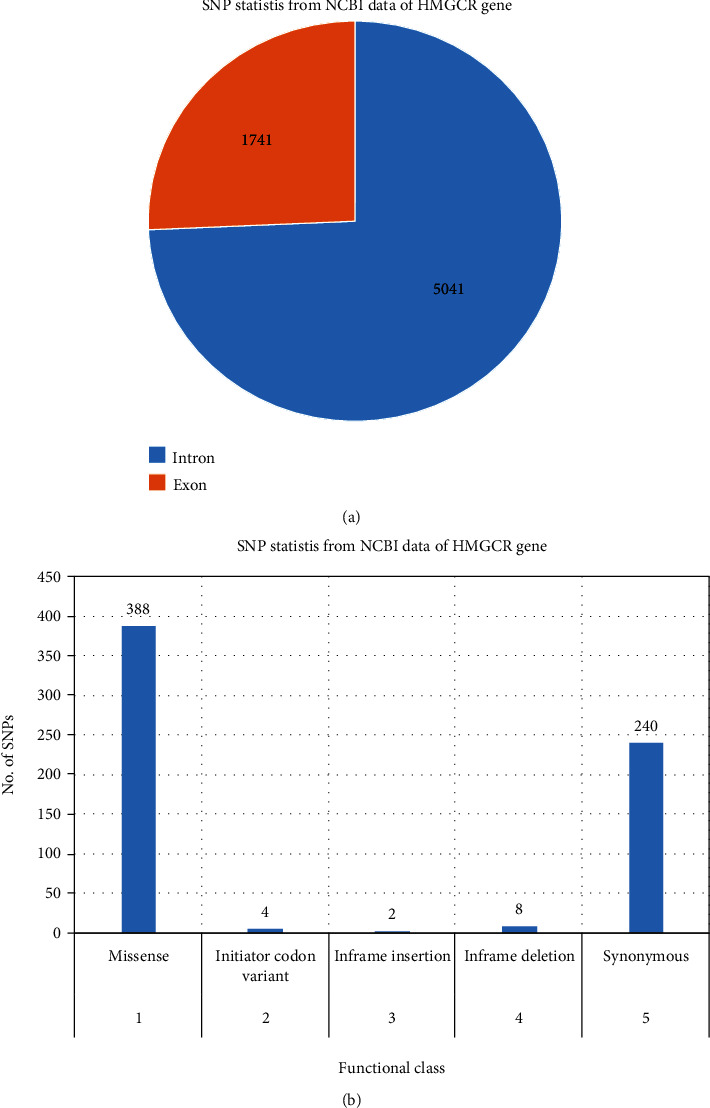
Major functional classes of HMGCR gene polymorphisms. (a) Functional class (intron and exon) of HMGCR gene polymorphisms. (b) Functional class (missense, initiator variant, inframe insertion, inframe deletion, synonymous) of HMGCR gene polymorphisms.

The HMGCR gene (25783 bp) consists of 23 exons. The SNP data for the HMGCR gene were collected from dbSNP as it contains the largest polymorphism database, despite housing both validated and nonvalidated polymorphism information [22]. The dbSNP contains a total of 6815 SNPs for the gene HMGCR where 388 SNPs were missense SNPs (Figure 2). Among 388 submitted missense SNP rsIDs from dbSNP, SIFT analyzed 7 missense SNPs to bear a deleterious effect with TI score ≤ 0.05; results are shown in Table 1. The corresponding 7 missense SNPs rs112503211, rs113949962, rs147043821, rs147818666, rs148335635, rs193026499, and rs368129510 had the tolerance index 0.1 and considered as damaging in the HMGCR gene (Table 1).

### 3.2. Coding Missense SNPs rs147043821 and rs193026499 are the Two Most Probable Damaging Mutations in HMGCR

The PolyPhen program was used to determine the missense SNPs with the potential to cause structural modifications due to the amino acid substitution. A total of 388 missense SNP rsIDs were submitted to the PolyPhen server, and in the resulting output, 27 amino acid substitutions have been reported to be probably damaging with a PSIC score ranging from 0.539 to 1. Seven missense SNPs (rs112503211, rs113949962, rs147043821, rs147818666, rs148335635, rs193026499, and rs368129510) were identified by SIFT as deleterious, also marked to be damaging by the PolyPhen-2 program as well (Table 2).

**Figure 3:**
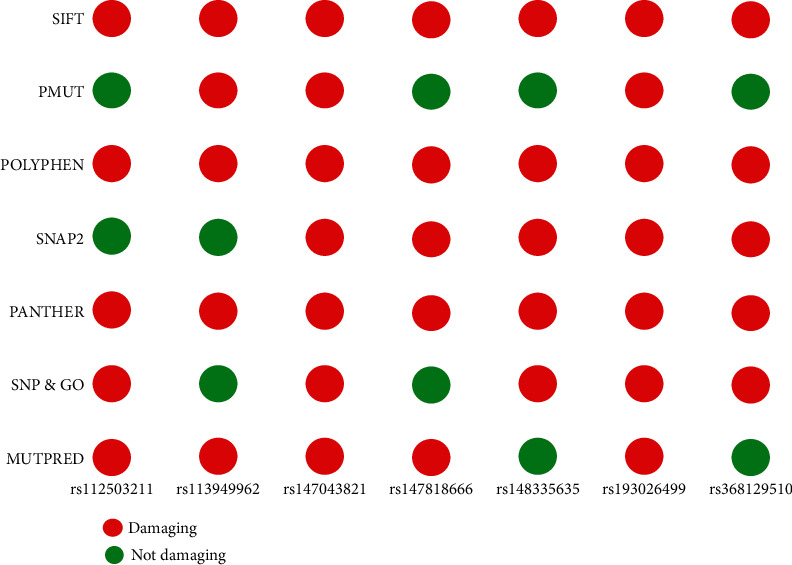
Damagicity of the functional missense SNP polymorphism.

To further validate the results of the tools used beforehand, we analyzed the missense SNPs with the following in silico SNP prediction algorithms: PMUT, SNAP, PANTHER, MUTPRED, and SNP&GO. The missense SNPs which are marked as deleterious by both SIFT and PolyPhen-2 server were principally selected. The results generated from the abovementioned tools were further combined and compared with the result of SIFT and PolyPhen server. In the combined results of 388 missense SNPs, only 7 (rs114166108, rs113949962, rs182539049, rs145415894, rs142939718, rs35896902, and rs150721457) were predicted as disease related by at least 5 out of the 7 tools (Figure 3). Two missense SNPs, rs147043821 and rs193026499, showed positive results in all the 7 tools (Tables 3–7).

### 3.3. High-Risk Missense SNPs are Located in the Conserved Region

**Figure 4:**
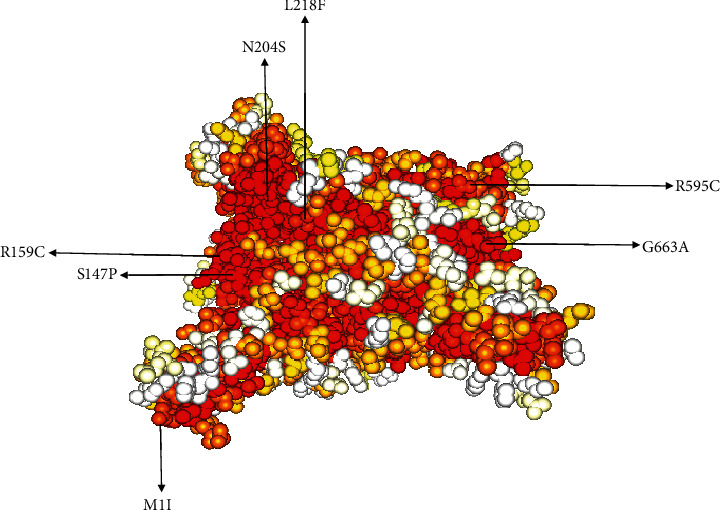
Conservation profile of functional missense SNPs. Map the conservation index as calculated by the Rate4Site algorithm. Color code is a gradient from red (more conserved) to white (more diverse) through yellow (mild conservation).

Biological processes rely on functional sites of proteins such as catalytic sites, allosteric sites, and protein-protein interaction sites. Amino acids present in these biologically active sites tend to be highly conserved, compared to any other residues in the protein. Any substitution of these residues generally leads to complete loss of biological functions and renders severe damaging effect to the biological process itself [25]. The retrieved amino acid sequence corresponding to missense SNPs was utilized to identify the suitable template to build the 3D structure. To predict the 3D structure, retrieved amino acid sequence was submitted to the NCBI protein BLAST tool to recognize the structure of the closest related proteins. The structure PDB ID: 3cd5 with 99.8% identity was selected and built 3D structure. InterEvDock2 identifies putative structural and functional residues and determines their evolutionary conservation [26]. Although a complete analysis was done, we focused on the conservation profile of the selected 7 high-risk missense SNP locations. The analysis showed that residues S147, M1, L218, G663, N204, R595, and R159 are highly conserved (Figure 4). These conserved residues in HMGCR might have imperative functional importance and are identified as functional or structural based on their location relative to the protein surface or the protein core.

### 3.4. High-Risk Missense SNPs are Capable of Inducing Protein Unfolding

**Figure 5:**
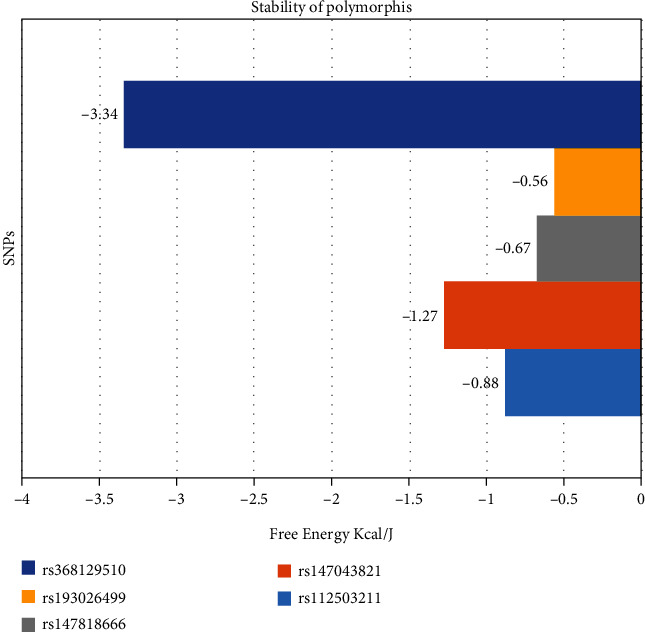
Free energy calculation of polymorphisms.

The neural network-based routine tool I-Mutant 2.0 was used for examining the potential modifications in protein stability due to mutations. Models with the following mutations S147, M1, L218, G663, N204, R595, and R159 were submitted to the server for DDG stability prediction and RSA calculation. All the mutations decreased protein stability except rs113949962, which is shown to be increasing structural stability (0.58 kcal/mol). Mutation rs368129510 accounted for the lowest DDG value (−3.34 kcal/mol), meaning to be more unstable due to this mutation (Figure 5). All other mutations rs112503211, rs147043821, rs147818666, and rs193026499 have the DDG values, respectively, -0.88 kcal/mol, -1.27 kcal/mol, -0.67 kcal/mol, and -0.56 kcal/mol; this suggests decreased protein stability, due to DDG values being less than 0 (Figure 5). Further, we have also analyzed the surface accessibility surface area (SASA) and angles of the protein structure of alpha helix and beta sheet in both wild and mutant models of HMGCR. All the mutations reside in alpha helix and beta sheet which can induce the conformational change in protein (Table 8).

SASA may change due to mutation when the amino acid substitution occurred. Here, the SASA value has been changed in all the mutations except rs147818666. The SASA value of rs368129510 has been slightly decreased than the wild-type model. The increasing SASA value of the mutations rs147043821 and rs193026499 may lead to the unfolding of the protein 3D structure, meaning the loss of the biological functions of the HMGCR gene (Table 8).

### 3.5. High-Risk Polymorphisms Are Likely to Alter the Domain Structures of HMGCR

**Figure 6:**
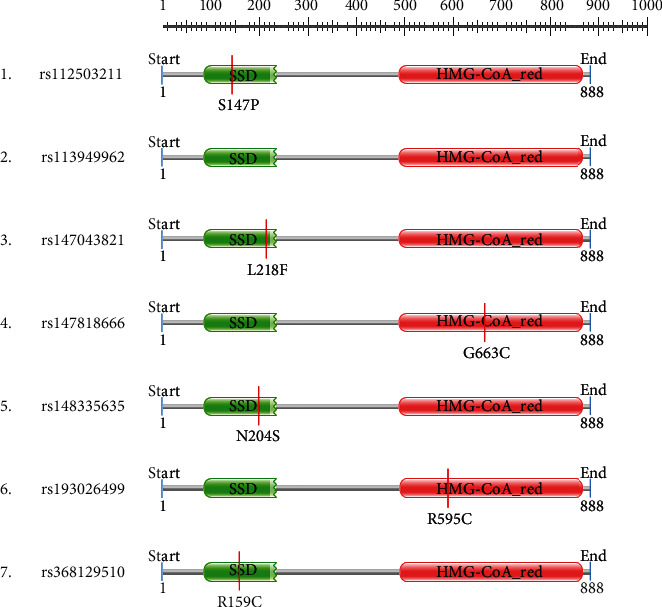
Domain association of high-risk polymorphism.

The PROSITE-ExPasy tool was used to search for domain structures in HMGCR and map the mutations in the domains for determining the changes they might cause in the domain structures. The tool searches the UNIProtKB database for motifs and in the produced result showed sterol-sensing domain (SSD) and hydroxymethylglutaryl-coenzyme A reductase (HMG-Co-A) domain in HMGCR. The SSD domain consists of 61–218 amino acid regions in the HMGCR gene, and HMG-Co-A is composed of 464–871 amino acid residues in the HMGCR region. All mutations S147, L218, G663, N204, R595, and R159C except 1MI are located in the SSD and HMG-Co-A domain (Figure 6).

### 3.6. Molecular Dynamic Simulation

**Figure 7:**
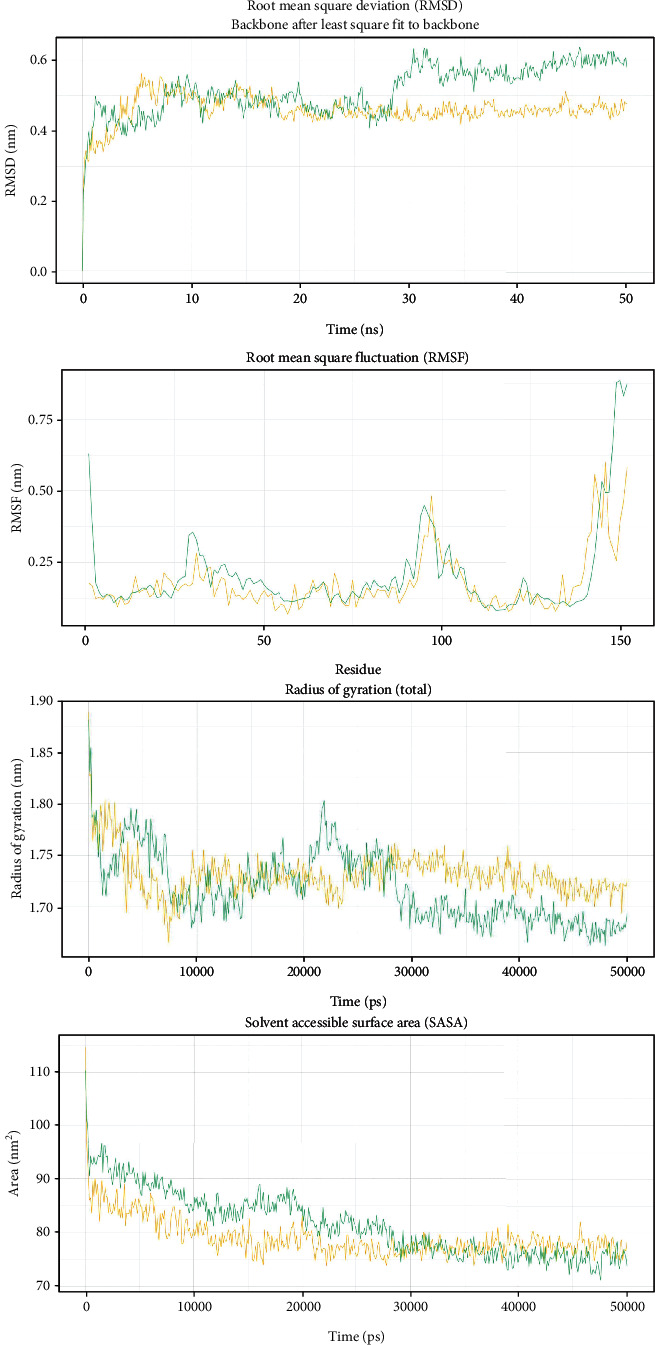
RMSD, RMSF, Rg, and SASA analysis of wild-type HMGCR (yellow) and the mutant rs147043821 (green).

**Figure 8:**
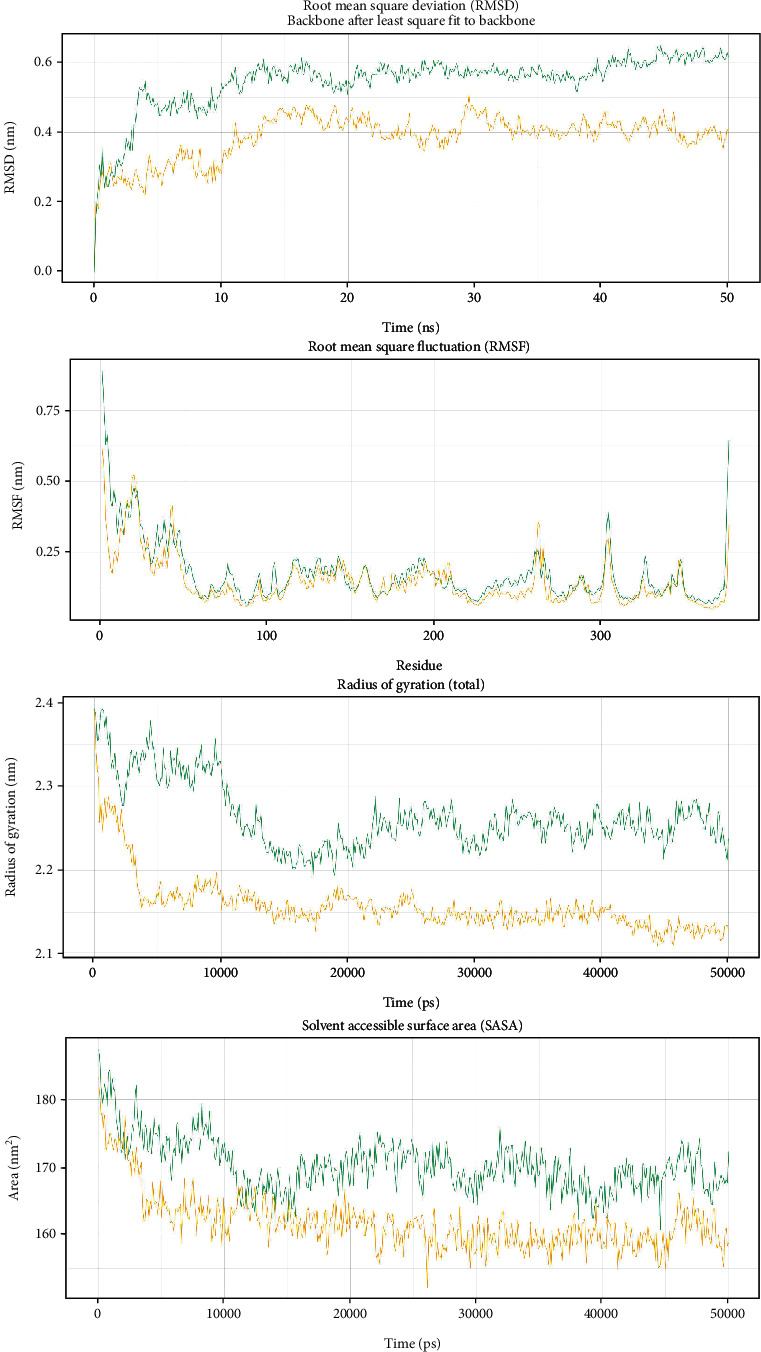
RMSD, RMSF, Rg, and SASA analysis of wild-type HMGCR (yellow) and the mutant rs193026499 (green).

Following 50 ns molecular dynamic simulation, RMSD, RMSF, Rg, and SASA calculations were carried out for the SNPs rs147043821 (Figure 7) and rs193026499 (Figure 8). RMSD analysis revealed that both rs147043821 and rs193026499 had a destabilizing effect on HMGCR. In both cases, the mutant exhibited higher structural deviation compared to the wild-type protein. Both the mutants rs147043821 and rs193026499 also showed regional flexibility as uncovered by the RMSF values. In terms of compactness, the SNP rs193026499 showed marked difference from its wild counterpart. The mutant remained in an unfolded state throughout the simulation while the wild type maintained a folded conformation. For the mutant rs147043821 however, the observed change in radius of gyration was not that drastic. SASA calculations indicated that the mutant rs193026499 introduced susceptibility to disruption by solvents. The mutant rs147043821 also followed the same trend, but its susceptibility became equivalent to the wild type near the end of the simulation.

## 4. Discussion

Single nucleotide polymorphisms (SNPs) are the main cause of most genetic diseases, because more than half of known genetic disorders involve amino acid substitutions. SNPs are exceptional genetic markers and play an important role in disease research because they are dispersed throughout the entire human genome. Although some disease-related SNPs are found in exons or coding regions, there are also SNPs that appear in the intronic regions of genes and interfere with regulatory regions, which in turn affect the splicing process and gene expression. Population-based surveys have become tough with the growing number of reported and recorded SNPs; utilizing the sheer number of SNPs data makes it demanding to choose a target for scrutiny which are most likely to contribute to disease development. The dry lab method is a convenient way in these circumstances to distinguish the deleterious SNPs using dedicated algorithms that can discriminate between neutral and deleterious SNPs by examining the databases and combining functional and structural evidence about the ultimate effect of a polymorphism.

The search for missense SNPs in dbSNP against HMGCR resulted in 6815 hits. The rsIDs of which were queried into SIFT and PolyPhen-2 servers. From the total SNPs of HMGCR, we selected only missense type of polymorphism for further investigation as it can have an effect on protein structure. We analyzed 388 missense polymorphisms by employing seven tools to observe how deleterious effect they can exert upon the protein structure (Figure 2). Seven SNPs were predicted deleterious in common in both SIFT and recent studies have found several accounts indicating HMGCR linkage to cholesterol biosynthesis, but there remains a significant amount of polymorphism data on HMGCR that awaits extensive population-based and clinical studies. In this current study, the SNP databases were analyzed to find out SNPs that might potentially be deleterious for HMGCR through the employment of computational methods, Polyphen-2 algorithms. Furthermore, we analyzed the data with several other SNP analyzing algorithms, and in a combined result, also 7 missense SNPs were predicted deleterious. We have employed SIFT, PolyPhen, PMUT, SNAP, PANTHER, MUTPRED, and SNP&GO algorithms to characterize the 388 missense SNPs found available in the database. However, only seven polymorphisms rs112503211, rs113949962, rs147043821, rs147818666, rs148335635, rs193026499, and rs368129510 have shown the deleterious effect upon the protein structure (Figure 3, Tables 1–7). We have employed the InterEvDock2 server to find out the conserveness of the wild residues. We have found these residues are in the conserved region, and if these residues alter, the protein structure would be hampered to initiate its normal activity (Figure 4). The predicted SNPs are located in the SSD and HMG-CoA domains of the protein, which ultimately may hamper the biological activity of HMGCR. Mutations in SSD could be a barrier to sense the sterol for cholesterol regulation into our body (Figure 6). For further analysis, we have explored the protein data bank that contained a structure (3cd5) with 99.8% identity with HMGCR. We performed a free energy calculation of the mutant and wild-type models. The free energy of all the mutated models declined considerably from the wild-type models. We have found all the selected polymorphisms S147, M1, L218, G663, N204, R595, and R159 have shown the protein destability upon the polymorphism except M1 (Figure 5). Further, we have utilized the STRIDE server by which we have observed the structural variation of the mutant models. A mutation may alter the folding pattern of the protein due to change in amino acid with another. Therefore, protein function can be lost ultimately prompting the disease condition. The selected polymorphisms rs112503211 (S147P), rs147043821 (L218F), rs193026499 (R595C), and rs368129510 (R159C) changed the solvent accessible surface area (SASA) and phi and psi angle than the wild-type models. The polymorphism rs147818666 (G663A) did not show any changes in the mutant model. It is to be noted that rs368129510 (R159C) and rs147043821 (L218F) changed in the alpha helix motif whereas rs193026499 (R595C) showed the changes in the beta sheet motif. Therefore, these three polymorphisms rs368129510 (R159C), rs147043821 (L218F), and rs193026499 (R595C) might change the folding pattern and stability of the protein. From the 50 ns molecular dynamic simulation carried out in GROMACS for wild-type HMGCR, rs147043821 and rs193026499, it is clear that these two SNPs have the ability to exert a significant destabilizing effect on the protein and thus disrupt its function (Figures 7 and 8).

In the process of this study, it was observed that despite some correct assumption, the web-based tools need to be more precise in detecting deleterious SNPs and population-based studies are essential to recognize and examine the predicted SNPs in different populations. By cross-referencing all the data from the seven algorithms, we observed two SNPs rs147043821 and rs193026499 have shown the damaging effect in all seven algorithms; therefore, these two could be the most promising polymorphism in the HMGCR.

**Figure 9:**
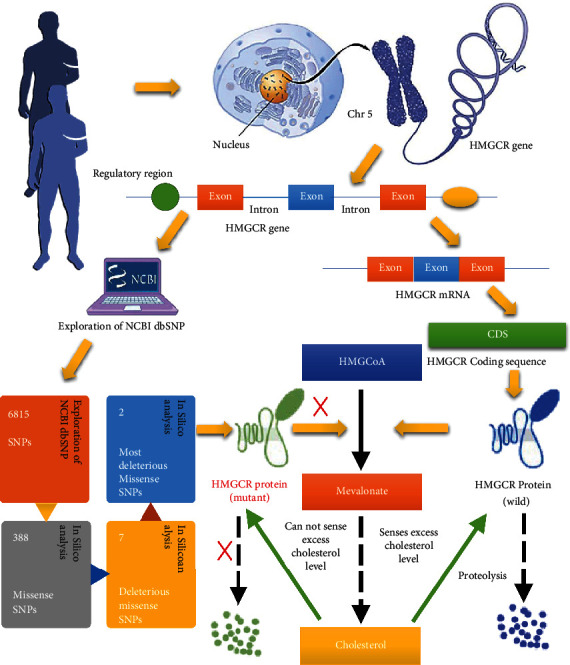
Hypercholesterolemia process by polymorphisms.

HMGCR is embedded in the endoplasmic reticulum (ER) membrane. It consists of two distinct domains: N-terminal domain or sterol-sensing domain (SSD) and C-terminal domain or catalytic domain. SSD anchors the protein in ER membrane that senses sterol or cholesterol level in the cell. It either directly or indirectly can sense the intracellular level of sterol/cholesterol. The catalytic domain is protruded in the cytosol and contains all the catalytic activities of HMGCR. The SSD of HMGCR senses the excess level of sterol/cholesterol in the cell, and the sensing result might induce a conformational change in SSD. This causes the protein susceptible to rapid sterol-induced degradation. Whenever there is enough cholesterol present in the cell, HMGCR undergoes proteolysis, and thereby, the quantity of HMGCR is decreased. When SNPs are present in SSD, it may fail to sense intracellular cholesterol levels; as a result, proteolysis of HMGCR does not occur. When present in the catalytic domain, SSD can sense cholesterol level but the further steps of endoplasmic reticulum-associated degradation (ERAD) may be hampered, and hence, catalytic domains fail to be degraded. So, rs147043821 in SSD or rs193026499 in the catalytic domain of HMGCR would hamper feedback regulation and upsurge the hypercholesterolemia, which might be a great concern for health (Figure 9). Though the findings of this study suggest that some SNPs, such as rs147043821 and rs193026499, may cause damage to the HMGCR protein, it is strongly advised that the mutational effect on the structure should be investigated in a wet lab experiment before any conclusive confirmation of their usefulness.

## 5. Conclusion

The present study analyzed the SNPs of HMGCR gene and predicted seven deleterious SNPs, viz., rs112503211, rs113949962, rs147043821, rs147818666, rs148335635, rs193026499, and rs368129510 through SNP analyzing tools. Among them, rs147043821 and rs193026499 are most likely to have the detrimental effect in the HMGCR gene. Molecular dynamic study also showed that the presence of these two SNPs in the HMGCR gene could have the destabilizing effect in the structure; thus, these SNPs could disrupt the functional effect of the HMGCR gene. Therefore, the results of this study might increase the risk of cholesterol biosynthesis in the human body. However, after the in silico discoveries, wet lab confirmation is required.

## Figures and Tables

**Table 1:** Impact of amino acid substitution on protein function using the SIFT.

SI	SNP	Ref allele	Alt allele	Amino acid change	Gene ID	Transcript ID	Protein ID	Region	Sift prediction
1	rs112503211	T	C	S147P	ENSG00000113161	ENST00000287936	ENSP00000287936	CDS	Deleterious
2	rs113949962	G	A	M1I	ENSG00000113161	ENST00000287936	ENSP00000287936	CDS	Deleterious
3	rs147043821	G	C	L218F	ENSG00000113161	ENST00000287936	ENSP00000287936	CDS	Deleterious
4	rs147818666	G	C	G663A	ENSG00000113161	ENST00000287936	ENSP00000287936	CDS	Deleterious
5	rs148335635	A	G	N204S	ENSG00000113161	ENST00000287936	ENSP00000287936	CDS	Deleterious
6	rs193026499	C	T	R595C	ENSG00000113161	ENST00000287936	ENSP00000287936	CDS	Deleterious
7	rs368129510	C	T	R159C	ENSG00000113161	ENST00000287936	ENSP00000287936	CDS	Deleterious

**Table 2:** Functional characterization of missense SNPs by PolyPhen.

SI	SNP	Protein acc	Position	AA1	AA2	Prediction/confidence			
						Probability	HumDiv	Probability	HumVar
1	rs112503211	P04035	147	S	P	Probably damaging	Score: 1.000 Sensitivity: 0.00 Specificity: 1.00	Probably damaging	Score: 0.993 Sensitivity: 0.47 Specificity: 0.96
2	rs113949962	P04035	1	M	I	Probably damaging	Score: 0.97 Sensitivity: 0.77 Specificity: 0.95	Possibly damaging	Score: 0.650 Sensitivity: 0.79 Specificity: 0.84
3	rs147043821	P04035	218	L	F	Probably damaging	Score: 1.000 Sensitivity: 0.00 Specificity: 1.00	Probably damaging	Score: 1.000 Sensitivity: 0.00 Specificity: 1.00
4	rs147818666	P04035	663	G	A	Possibly damaging	Score: 0.659 Sensitivity: 0.86 Specificity: 0.91	Possibly damaging	Score: 0.519 Sensitivity: 0.82 Specificity: 0.81
5	rs148335635	P04035	204	N	S	Probably damaging	Score: 1.000 Sensitivity: 0.00 Specificity: 1.00	Probably damaging	Score: 0.999 Sensitivity: 0.09 Specificity: 0.99
6	rs193026499	P04035	595	R	C	Probably damaging	Score: 0.999 Sensitivity: 0.14 Specificity: 0.99	Probably damaging	Score: 0.983 Sensitivity: 0.56 Specificity: 0.94
7	rs368129510	P04035	159	R	C	Probably damaging	Score: 1.000 Sensitivity: 0.00 Specificity: 1.00	Probably damaging	Score: 0.939 Sensitivity: 0.66 Specificity: 0.91

**Table 3:** Disease association study of missense SNPs by PMUT.

SI	SNP	Protein	Position	Mutation	Prediction
1	rs112503211	P04035	147	S → P (Ser → Pro)	0.48 (83%) neutral
2	rs113949962	P04035	1	M → I (Met → Ile)	0.60 (83%) disease
3	rs147043821	P04035	218	L → F (Leu → Phe)	0.55 (81%) disease
4	rs147818666	P04035	663	G → A (Gly → Ala)	0.43 (85%) neutral
5	rs148335635	P04035	204	N → S (Asn → Ser)	0.48 (83%) neutral
6	rs193026499	P04035	595	R → C (Arg → Cys)	0.79 (89%) disease
7	rs368129510	P04035	159	R → C (Arg → Cys)	0.48 (83%) neutral

**Table 4:** Prediction of damaging effect of SNP in HMGCR gene by SNAP2.

SI	SNP	Wild-type amino acid	Position	Variant amino acid	Predicted effect	Score	Expected accuracy
1	rs112503211	S	147	P	Neutral	-22	61%
2	rs113949962	M	1	I	Neutral	-27	61%
3	rs147043821	L	218	F	Effect	12	59%
4	rs147818666	G	663	A	Effect	5	53%
5	rs148335635	N	204	S	Effect	48	71%
6	rs193026499	R	595	C	Effect	47	71%
7	rs368129510	R	159	C	Effect	73	85%

**Table 5:** Damagicity prediction of polymorphism by PANTHER.

SI	SNP	Substitution	Preservation time (millions of years)	Message
1	rs112503211	S147P	673	Probably damaging
2	rs113949962	M1I	3807	Probably damaging
3	rs147043821	L218F	673	Probably damaging
4	rs147818666	G663A	3806	Probably damaging
5	rs148335635	N204S	673	Probably damaging
6	rs193026499	R595C	673	Probably damaging
7	rs368129510	R159C	673	Probably damaging

**Table 6:** Effect in functional motif in HMGCR gene by MUTPRED tool.

SI	SNP	Substitution	MutPred2 score	Affected PROSITE and ELM motifs	Molecular mechanisms with *P* values ≤ 0.05
1	rs112503211	S147P	0.652	ELME000063, ELME000064, ELME000136, ELME000159, ELME000202, ELME000239, ELME000249, ELME000	Gain of phosphorylation at S146, Prob: 0.29, *P* value: 0.02 Altered transmembrane protein, Prob: 0.17, *P* value: 9.1*e* − 03 Loss of ubiquitylation at K142, Prob: 0.16, *P* value: 0.03 Loss of GPI-anchor amidation at N148, Prob: 0.03, *P* value: 8.4*e* − 03
2	rs113949962	M1I	0.881	ELME000355	Altered disordered interface, Prob: 0.39, *P* value: 5.1*e* − 03 Altered ordered interface, Prob: 0.27, *P* value: 5.6*e* − 03 Altered signal peptide, Prob: 0.18, *P* value: 8.8*e* − 04 Loss of N-terminal acetylation at M1, Prob: 0.03, *P* value: 5.6*e* − 03
3	rs147043821	L218F	0.781	ELME000239, ELME000333, ELME000335	Loss of helix, Prob: 0.33, *P* value: 1.2*e* − 03 Gain of strand, Prob: 0.28, *P* value: 7.5*e* − 03
4	rs147818666	G663A	0.856	ELME000063, PS00008	Gain of helix, Prob: 0.28, *P* value: 0.03; loss of allosteric site at M659, Prob: 0.24, *P* value: 0.02; loss of acetylation at K662, Prob: 0.24, *P* value: 0.02
5	rs193026499	R595C	0.569	ELME000155	Altered ordered interface, Prob: 0.31, *P* value: 0.01 Gain of allosteric site at R590, Prob: 0.24, *P* value: 0.01 Loss of catalytic site at R590, Prob: 0.22, *P* value: 8.8*e* − 03 Gain of ADP-ribosylation at R598, Prob: 0.19, *P* value: 0.04 Altered transmembrane protein, Prob: 0.13, *P* value: 0.02 Altered metal binding, Prob: 0.05, *P* value: 0.04
6	rs368129510	R159C	0.304	—	—

**Table 7:** Disease probability prediction of SNP by SNP&GO.

SI	SNP	Mutation	Prediction	Reliability index (RI)	Probability	Method
1	rs112503211	S147P	Disease	4	0.710	PhD-SNP: F[S] = 52%, F[P] = 0%, Nali = 66
2	rs113949962	M1I	Neutral	3	0.352	PhD-SNP: F[M] = 100%, F[I] = 0%, Nali = 37
3	rs147043821	L218F	Disease	4	0.713	PhD-SNP: F[L] = 91%, F[F] = 0%, Nali = 66
4	rs147818666	G663A	Neutral	6	0.197	PhD-SNP: F[G] = 62%, F[A] = 33%, Nali = 377
5	rs148335635	N204S	Disease	6	0.820	PhD-SNP: F[N] = 75%, F[S] = 0%, Nali = 66
6	rs193026499	R595C	Disease	6	0.793	PhD-SNP: F[R] = 51%, F[C] = 0%, Nali = 356
7	rs368129510	R159C	Disease	6	0.806	PhD-SNP: F[R] = 52%, F[C] = 0%, Nali = 66

**Table 8:** Structural variation upon the mutational impact.

SI	Mutation	Mutation type	Residue name and number	Structure	SASA	Phi angle	Psi angle
1	rs112503211	Wild	S147	Coil	15.4	-57.58	144.73
		Mutant	147P		18.8	-60.07	140.74
2	rs368129510	Wild	R159	Alpha helix	85.6	-57.10	-39.91
		Mutant	159C	Alpha helix	68.3	-66.57	-41.71
3	rs147043821	Wild	L218	Alpha helix	21.4	-65.25	-40.28
		Mutant	218F	Alpha helix	78.2	-64.22	-44.63
4	rs193026499	Wild	R595	Beta sheet	2.5	125.89	145.20
		Mutant	595C	Beta sheet	67.7	126.90	144.29
5	rs147818666	Wild	G663	Alpha helix	0.0	-64.63	-39.48
		Mutant	663A	Alpha helix	0.0	-64.96	-39.53

## Data Availability

The datasets analyzed during the current study are available from the corresponding author on reasonable request.

## Abbreviations

HMGCR: 3-Hydroxy-3-methylglutaryl-CoA reductase
SNPs: Single nucleotide polymorphisms
LDL: Low-density lipoprotein
GWAS: Genome-wide association studies
CVD: Cardiovascular disease
GO: Gene Ontology
SASA: Surface accessibility surface area
SSD: Sterol-sensing domain.
